# An examination of the effects of a patient-designed-and-informed participant information sheet in comparison with a standard, researcher-designed information sheet on recruitment, retention and understanding: Protocol for a study-within-a-trial

**DOI:** 10.12688/hrbopenres.12981.2

**Published:** 2020-03-30

**Authors:** Christopher P. Dwyer, Robert A. Joyce, Eimear M. Bane, Anusha Moses, Alberto Alvarez-Iglesias, Sinéad M. Hynes

**Affiliations:** 1 Discipline of Occupational Therapy, School of Health Sciences, National University of Ireland, Galway, Galway, Ireland; 2 Health Research Board Clinical Research Facility, National University of Ireland, Galway, Galway, Ireland

**Keywords:** Participation information, recruitment, retention, multiple sclerosis, public and patient involvement, PPI, SWAT

## Abstract

**Background:** This protocol describes a double-blind, randomised non-inferiority study-within-a-trial (SWAT), comparing the effects of a patient-designed-and-informed participant information sheet with a standard, researcher-designed participant information sheet on recruitment, retention, decision certainty, participant information sheet understanding and likeability. The SWAT is part of a larger trial that aims to evaluate the feasibility and preliminary efficacy of a cognitive occupation-based programme for people with MS (COB-MS) on cognitive and daily functioning for people with multiple sclerosis.

**Methods**: During the study, 120 people with multiple sclerosis will be randomly allocated to one of the two groups, where they will either receive a standard participant information sheet or a patient-designed participant information sheet. Recruitment and retention will be analysed, as well as decision certainty, likability and understanding.

**Discussion: **Results will provide recommendations for recruitment, consent and retention for future trials, as well as shed some light on the factors influencing the understanding and likeability of a trial’s participant information sheet. Recommendations will also be made regarding patient and public involvement in developing and/or aiding the development of participant information sheets.

**Registration**: SWAT: Northern Ireland Hub for Trials Methodology Research SWAT Repository Store ( SWAT105). COB-MS trial: ISRCTN11462710.

## Introduction

### Background and rationale

Recruitment is a critical process within research interventions, given its impact on statistical power, the validity of findings and investment of resources ( Britton *et al*., 1998; Wade *et al*., 2009). Apart from advertisement materials, the participant information sheet (PIS) is the primary source of written information that potential participants engage with during the recruitment process. Indeed, it is crucial to ensure that potential participants understand both the broad and specific implications of the study to which they are consenting ( McCaughey *et al*., 2016). Though provision of a PIS is requisite as part of the informed consent process, in which case the PIS itself has been subject to ethical review, such ethical consideration does not necessarily account for *quality* of the PIS. Thus, it can be argued that just because the presentation of a PIS is not unethical, this does not ensure that it is appropriate.

Research indicates that understanding of the PIS is often poor amongst participants in health-related research ( Khan *et al*., 2014; McCaughey *et al*., 2016). However, only a limited body of research has evaluated the effects of various manipulations to PIS development on recruitment and comprehension, yielding mixed results (e.g. Cockayne *et al*., 2017; Knapp *et al*., 2011; Manley *et al*., 2015). As some of these manipulations can be costly with respect to both finances and time, future research is necessary to identify a practical, feasible means of enhancing PIS clarity and comprehension, as well as subsequent participant retention.

Having PIS design input from an individual eligible to participate in the intervention (e.g. living with the chronic illness), but without any personal bias involved with actually participating, may yield positive effects on recruitment and comprehension - that is, PIS development led by a *public and patient involvement* (PPI) member of the research team (not from a research background), who would otherwise be eligible to participate in the intervention. PPI is an effective means of enhancing the likelihood of a successful trial by involving people with lived experience of a particular condition as partners throughout the research process ( Crocker *et al*., 2018). In light of extant theory and research, a PIS developed in light of PPI may enhance trial understanding and recruitment (with respect to consent), as well as participant retention.

### Objective

The objective of the current research (i.e. a study-within-a-trial; SWAT) is to compare the effects of two PISs designed to facilitate informed consent of potential participants – a (patient) PPI-designed-and-informed PIS in comparison and a standard, researcher-designed PIS on: recruitment, decision certainty, retention; understanding, readability, accessibility, likeability and decision to consent. The SWAT is part of a larger trial that aims to evaluate the feasibility and preliminary efficacy of an eight-session cognitive occupation-based programme for people with MS (COB-MS) on cognitive and daily functioning for people with MS (PwMS).

## Methodology

### Ethical statement

Ethical approval was awarded by Galway University Hospitals on 13.08.2019 Ref: C.A 2231 and the study will be conducted at the National University of Ireland, Galway. All participants will take part in this study based on informed consent, in which they know their non-personalised data will be reported in published dissemination.

### Trial design

The current SWAT is a double-blind, randomised non-inferiority trial comparing the effects of a patient-designed-and-informed PIS with a standard, researcher-designed PIS on recruitment, decision certainty, participant retention, PIS understanding, PIS likeability and decision to consent. Both PISs are designed specifically for an MS cohort. The SWAT will be conducted in the context of a single-blind, cluster-randomised controlled feasibility and preliminary efficacy trial of the eight session COB-MS programme – a Cognitive Occupation-Based programme for people living with MS – in comparison with a treatment as usual, wait-list control group (i.e. the main trial). For further details of the study within which this SWAT will be conducted (from here on referred to as the ‘main trial’), see ISRCTN11462710.

### Study setting

This is a community-based research study. Data will be collected in Ireland. The main study site is at NUI Galway; but data will be collected nationwide, dependant on the location of the participants.

### Interventions

Two PISs were developed. A *standard, researcher-designed PIS* (SRPIS) was written by the post-doctoral researcher – from both the SWAT and the main trial – who has over 10 years’ research experience. The researcher wrote the PIS in light of templates from past trials for structure, making sure to include/address study background, procedure, eligibility, consent, funding/support and descriptions of both potential risks and benefits. A *PPI-designed-and-informed PIS* (PPIPIS) was developed by a PPI member of the research team - who was neither from a research or medical background, nor had experience in these fields - who would otherwise be eligible to participate in the intervention. The PPI member wrote the PIS from a patient perspective, in light of what was deemed both necessary for potential participants to know and useful to know. The only restriction on PPIPIS development was that the PPI member was required to include/address study background, procedure, eligibility, consent, funding/support and descriptions of both potential risks and benefits. Following development of the PPIPIS, the document was further analysed, evaluated and subsequently approved as appropriate by other PPI members through discussion and agreement within a PPI focus-group; specifically: one other PPI member from the trial steering committee and an external PPI consultation group which was convened to discuss issues related to outcome measures and recruitment material, such as this. Notably, the PIS developers were blinded to each other’s PIS and did not liaise or discuss the PISs during their development, both of which were submitted separately to the primary investigator (PI) for subsequent submission for ethical approval. Both PISs were accompanied by a PI-developed addendum regarding GDPR guidelines in order to ensure consistency in this context, for ethical purposes. See *Extended data*, for the two PISs, GDPR addendum and consent form ( Dwyer, 2020).

### Outcome measures

Recruitment will be measured dichotomously by whether or not the PwMS consented to participate in the main trial.

The Decisional Conflict Scale (DCS; O’Connor, 1995) is a 16 item questionnaire, answered via a five-point Likert scale, ranging from strongly agree (0) to strongly disagree (4), used to measure decision certainty, with respect to decision to provide consent to participate in this SWAT. The scale is established as valid and reliable with test–retest correlations and Cronbach’s alpha of 0.78 ( O’Connor, 2012).

Retention will be measured dichotomously by whether or not the PwMS completed participation in the trial. Notably, *level of retention* will also be measured by stages of completion, including (0–8) COB-MS sessions completed and (1–4) testing phases completed (i.e. on a scale of 1–12).

Understanding, readability, accessibility, likeability and decision to consent will be assessed via a six-item questionnaire, developed through discussion and agreement with a PPI focus-group, to be answered via six-point Likert scale, ranging from strongly disagree (0) to strongly agree (5):

1. The *Study Information Sheet* played a large role in my decision to participate in the study. (Decision to consent)
2. I was able to read the information presented in the *Study Information Sheet*. (Understanding)
3. I was able to understand the information presented in the *Study Information Sheet*. (Understanding)
4. The language used in the *Study Information Sheet* was accessible to me. (Understanding)
5. I knew I was going to consent participate before I was even presented the *Study Information Sheet*. (Decision to consent)
6. Overall, I liked *Study Information Sheet* that was presented to me. (Likeability)

### Sample size

**Figure 1.**
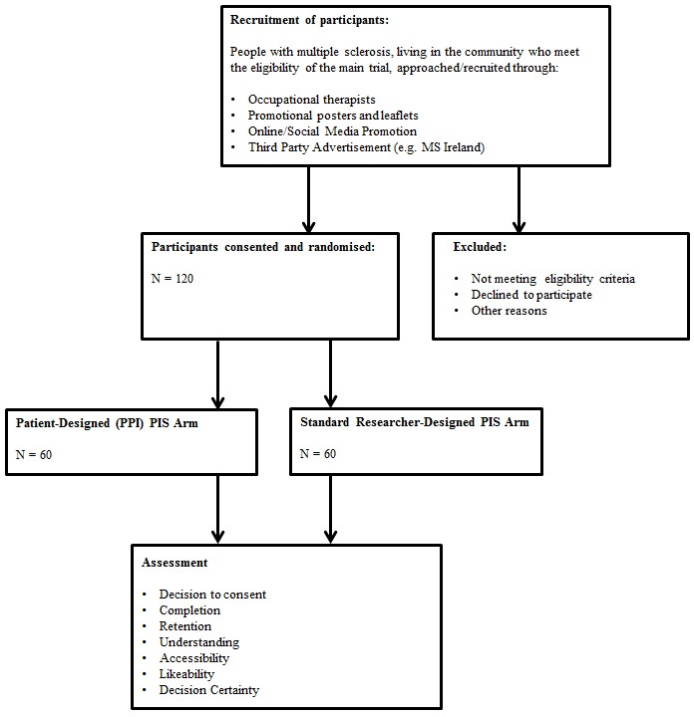
CONSORT flowchart of participants.

As the main trial is a feasibility study, a formal sample size calculation is not required. Instead, a pragmatic approach is adopted, based on an average recruitment rate for National Institute for Health Research (UK) funded randomised controlled trials (RCTs). A total of 120 PwMS will be recruited for the main trial; thus, the sample size for the SWAT will exceed 120 (i.e. accounting for ‘consent decliners’), until saturation has been reached. See Figure 1 for CONSORT flowchart of study participants.

### Recruitment

PwMS will be recruited through advertisement in relevant outlets, including newsletters and other publications (e.g. monthly *MS Ireland* newsletter), MS-related websites, discussion boards and forums (e.g. *MS Ireland*), recruited occupational therapists, posters in relevant clinics around the Republic of Ireland (e.g. GP, primary care clinics, physiotherapy and neurology), radio and social media. Advertisements (see *Extended data*; Dwyer, 2020) will not provide detailed trial information that would contaminate or influence assimilation via the PISs. Individuals interested in participating will self-select through contacting the researchers by email or phone; and informed consent will be obtained prior to participation.

### Interim analysis and stopping guidelines

There is no planned interim analysis. However, statistical analysis of descriptive data may be required in consideration of stopping guidelines for cases of unforeseen circumstances. Specifically, consistent with Avery *et al.* (2017), transparent reporting will be made around the decision-making process for stopping, amending or proceeding with the main trial and, likewise, the SWAT. As this is a SWAT embedded within a feasibility trial, stopping rules are distinct from those seen in a definitive trial and include two types of stopping guidelines – those specific unto the SWAT and those specific unto the main trial. The latter are relevant in this context, given that stopping the main trial prematurely would block assessment of participation completion and level of retention. The stopping rule specific unto the SWAT is recruitment of less than 70% during the recruitment phase set; protocol, including data collection period not tolerated by over 25% of participants. Stopping rules for the main trial also include: drop-out rate of participants during the COB-MS intervention is great than or equal to 50%; drop-out rate of occupational therapy participants greater or equal to 40%; serious adverse event(s) reported to the data monitoring committee (DMC) that are a direct result of the COB-MS and the DMC view require the stopping of the trial.

### Randomisation

PwMS participants will be randomly allocated to one of two study arms (i.e. SRPIS and a PPIPIS), using 1:1 allocation, via randomised block permutation (i.e. two randomised blocks of four and six per block). Randomisation will be conducted through a purpose-designed computer-generated system. Specifically, one researcher with statistical expertise will conduct the randomisation based on the sequence and type of randomisation described above. A second researcher, blind to the sequence, will collate the names of potential participants’ in light of a ‘first-come-first-serve’ basis regarding expression of interest in the study, and address information packs to potential participants based on the code yielded from the randomisation process. The code consists of a four digit, non-repeating string which cannot be ‘guessed’ by the blinded researcher. A third researcher will recode the data upon imputation, resulting in the blinding of the initial researcher, who will analyse the data.

### Inclusion and exclusion criteria

Inclusion and exclusion criteria are based on the main trial. Notably, however, though the inclusion/exclusion criteria are presented within the PISs, eligibility will not be confirmed until after completion of the SWAT. Thus, it cannot be ensured that participants will meet the main trial’s inclusion/exclusion criteria. Though the main trial’s criteria for eligibility are not relevant to the SWAT, records of eligibility and reasons for ineligibility will be recorded. Inclusion criteria for the main trial include:

- 1) aged 18 years of age or older;
- 2) fluent in written and spoken English;
- 3) have a diagnosis of multiple sclerosis (consistent with the McDonald Criteria for the Diagnosis of Multiple Sclerosis [ Thompson *et al*., 2018]);
- 4) cognitive difficulties, as shown by a score of >22 on the *Multiple Sclerosis Neuropsychological Screening Questionnaire* ( Benedict *et al*., 2004)
- 5) are clinically stable (i.e. not having an active relapse);
- 6) can provide informed consent;
- 7) no neurologic history other than MS, including evidence of current dementia;
- 8) no history of major depressive disorder, schizophrenia, or bipolar disorder I or II;
- 9) no history of diagnosed substance use or dependence disorder;
- 10) not currently undergoing any other form of cognitive rehabilitation; and
- 11) living in the community.

The exclusion criteria for the main trial are: (1) cognitive impairment that would affect reliable participation or capacity to give informed consent; (2) are incarcerated or institutionalized; and (3) significant neurological condition or organic brain damage (unrelated to MS).

### Procedure

Consistent with the recruitment strategy discussed above, all individuals interested in participating will self-select through contacting the researchers by phone or email. The duration of the recruitment period for both the main trial and SWAT is eight months. Potential participants will be sent a randomly allocated PIS in the post to consider before making their decision to participate in the main trial. Participants will also be sent a consent form for the main trial and the outcome measures relevant to this SWAT. Following verbal consent and screening via telephone, SWAT data and written consent will be collected by another member of the research team (i.e. an assistant psychologist trained in psychological assessment), who will have phoned in advance to organise a home visit for the main trial’s baseline assessment (i.e. within three months of receiving their randomly allocated PIS). Consenting participants may choose not to complete some aspects or all of the self-report SWAT outcome measures and, at the same time, remain in the main trial, in which case, partial response or non-response to the self-report SWAT outcome measures will be treated as missing data. Consistent with the protocol for the main trial, those who decline to participate in the trial will be asked, with informed consent, to provide the reasons why they have declined to participate in the main trial. These data will include the measures identified in this SWAT protocol. Those who consent to participate in this ‘decliner cohort’ will be asked to complete the measures already sent to them and return them completed to the research team. Those who do not consent to participate in the ‘decliner cohort’ will be thanked for their time and consideration. Rates of all forms of consent will be recorded. See Table 1 for SWAT schedule, consistent with SPIRIT guidelines.

**Table 1.** Study-within-a-trial schedule.

	Study period			
	Pre-intervention	Intervention	Post-intervention	Post-main trial
Timepoint	-t _1_	t _0_	t _1_	t _2_
**Self-selected enrolment**	X			
**Allocation**	X			
**Verbal consent**	X			
**Intervention**		X		
**Signed consent**			X	
**Assessments**				
*Recruitment*			X	
*Retention*				X
*Understanding*			X	
*Likeability*			X	
*Decision to consent*			X	
*Decision certainty*			X	

### Analysis

Statistical analysis will be conducted through two chi-square tests of independence, which will be performed to examine the relationship between source perspectives (i.e. SRPIS and PPIPIS) on both consent and trial completion (i.e. retention). A series of analyses of variance will be conducted to examine the effects of source perspective on level of retention, understanding, readability, accessibility, likeability, decision certainty and decision to consent regarding the two different PISs. Statistical significance will be determined at the .05 level. Descriptive statistics and correlations will be reported for all measures. Sub-group analyses will be conducted if warranted by the planned analyses to aid interpretation of the statistical findings (e.g. differences between: main trial completers and non-completers; or low and high scorers on the DCS).

### Data management and monitoring

A FAIR Data Management Plan ( Wilkinson *et al*., 2016) will be used for this SWAT, which ensures that all data are *findable*, *accessible*, *interoperable* and *reusable*. That is, collected anonymised data will be made openly available, where possible, in an ethical manner; and ensures that appropriate data management is conducted during all phases of the study. Upon collection, data will be imputed to an electronic file and stored on an encrypted, password-protected, hard drive. All hard copies of data will be kept securely in a locked cabinet at the study site. Confidentiality of all data and individual results will be protected at all times and anonymisation will be used throughout the study. Names or other personal identifiers will be securely stored separately from other data, identified by code, to ensure blinding. The statistician will analyse cleaned, depersonalised data. Blinded researchers, including the statistician, will only have access to cleaned, depersonalised data sets. Participants will be aware of and have consented to these processes in advance of participation. All data collection and storage will be conducted consistent with GDPR guidelines.

### PPI

PPI in research refers to the involvement of people with lived experience of a particular condition (e.g. MS) as partners throughout the research process and is often an effective means of enhancing the likelihood of a successful trial ( Crocker *et al*., 2018). Consistent with PPI practice, both the main trial and this SWAT have a PPI member as part of the research team for their entire durations. There are two PPI members on the trial steering committee and an external PPI consultation group has been convened to discuss issues – outcome measures and recruitment material. To reiterate, SWAT outcomes were in part developed through discussion and agreement with a PPI focus-group; and the one PPI research team member devised the PPI-developed PIS. Furthermore, both recruitment and dissemination of results will be aided through PPI through lay knowledge translation in the community.

### Safety

No harm is expected to come to participants from taking part in the SWAT. If any harm does occur, it will be recorded and reported to the Principal Investigator and relevant Ethics Committees.

### Study status

At the time of submission of this protocol, recruitment has commenced.

## Discussion

Early pilot studies have shown that COB-MS training may lead to improvements in daily living and cognitive functioning in people with MS ( Reilly & Hynes, 2018). However, these findings require replication with a larger participant pool; and, as such, any barriers to recruitment and retention should be avoided. Past RCTs have found recruitment of MS participants to be slow, with low uptake rates ( Carter *et al*., 2015; Cooper *et al*., 2011). Complicated or jargonistic PISs may hinder patient’s understanding of the study ( Parker *et al*., 2018). By involving people with MS in the design of MS-specific PISs, it may increase understanding and, in turn, both recruitment and retention ( Crocker *et al*., 2018). If a patient-informed PIS is found to be an effective way of increasing recruitment, then the current SWAT protocol could provide a beneficial template for future clinical research in MS. Overall, results from this SWAT may provide useful recommendations for recruitment, consent and retention for future trials, as well as shed some light on the factors influencing the understanding and likeability of a trial’s participant information sheet.

## Dissemination

Findings of the SWAT will be submitted for publication in a peer-reviewed journal and presented at both national (Ireland) and international conferences. This SWAT’s knowledge exchange plan also includes accessible outlets of dissemination for lay audiences, such as through PPI-oriented national meetings and other local level presentations and fora, social media, as well as NUI Galway communications, with an aim of targeting the research community and both PPI and funding bodies. Study results will be submitted for appropriate dissemination within six months of final data collection.

## Data availability

### Underlying data

No data are associated with this article.

### Extended data

Open Science Framework: An examination of the effects of a patient-designed-and-informed participant information sheet in comparison with a standard, researcher-designed information sheet on recruitment, retention and understanding: Protocol for a study-within-a-trial. https://doi.org/10.17605/OSF.IO/DGZBQ ( Dwyer, 2020)

This project contains the following extended data:

- PISs-GDPRAddendum-ConsentForm.docx (document containing patient information sheets, GDPR addendum and consent forms)
- TrialAdvertisements.docx (document containing advertisements for the trial)

Data are available under the terms of the Creative Commons Zero "No rights reserved" data waiver (CC0 1.0 Public domain dedication).

### Reporting guidelines

Open Science Framework: An examination of the effects of a patient-designed-and-informed participant information sheet in comparison with a standard, researcher-designed information sheet on recruitment, retention and understanding: Protocol for a study-within-a-trial. https://doi.org/10.17605/OSF.IO/DGZBQ ( Dwyer, 2020)
