# Supplementary material for: An examination of the effects of a patient-designed-and-informed participant information sheet in comparison with a standard, researcher-designed information sheet on recruitment, retention and understanding: Protocol for a study-within-a-trial
Source: HRB Open Res. 2020 Mar 30;3:4. Originally published 2020 Jan 24. [Version 2] doi: 10.12688/hrbopenres.12981.2 (PMC7140771; doi:10.12688/hrbopenres.12981.2)
Supplement: Peer review history

# Peer review: Reviewer response for version 2

**DOI:** 10.21956/hrbopenres.14116.r27279

**Published:** 2020-03-30

**Authors:** Vivian Welch

**Affiliations:** 1 School of Epidemiology and Public Health, Faculty of Medicine, University of Ottawa, Ottawa, Canada

Responses to peer review are excellent.

I confirm that I have read this submission and believe that I have an appropriate level of expertise to confirm that it is of an acceptable scientific standard.

# Peer review: Reviewer response for version 1

**DOI:** 10.21956/hrbopenres.14067.r27169

**Published:** 2020-03-09

**Authors:** Erik Lundstrom, Eva Isaksson

**Affiliations:** 1 Uppsala University, Uppsala, Sweden; 2 Karolinska Institutet, Solna, Sweden

**General comments**

 This manuscript addresses an important field: improvement of the patient information sheet to increase recruitment and retention in clinical trials. It is obvious that the authors know their field, given the information in the introduction and the length of the manuscript.

 My main concern is that it is not clear what the primary outcome is? See methods.

Overview

- The manuscript would be much better if it could be shorter, especially the introduction.
- Don't use so many abbreviations.
- I can’t find the two participant information sheet (PIS) (am I missing something?). It would be good to find a link early in the text.
- Generalisability - can the result of this study be used in other study groups or other diagnosis? 
- How is the cognitive status of this patients? Can this affect the result of this study or the content in the PIS?
- Did the PPI member that were designing the PPIPS had MS so that they could relate to specific questions and feelings in the information? That part is not clear. Maybe you should describe it more thoroughly.

Title

 A little wordy. Is it possible to shorten? 

 Maybe: Comparing standard information with patient-designed sheet: Protocol for a study-within-a-trial.

Abstract

 Background: It is a little hard to know how large the larger study is, just adding n=… would help. “The SWAT is part of a larger trial (n=…)”

 "Methods: During the study, 120 people with multiple sclerosis will be randomly allocated to one of the two groups, where they will either receive a standard participant information sheet or a patient-designed participant information sheet.”

 Question: Are they randomly allocated 1:1?

 "Recruitment and retention will be analysed, as well as decision certainty, likability and understanding." 

 Question: What is the primary outcome? How are the recruitment analysed? And I wonder, would you really expect higher recruitment and retention? I would be surprised if you get +2-3% more recruitment.

 "Discussion: Results will provide recommendations for recruitment, consent and retention for future trials, as well as shed some light on the factors influencing the understanding and likeability of a trial’s participant information sheet."

 Question: This is a very strong statement. It might provide recommendations on recruitment. But also on consent, and retention? 

Introduction

 It is obvious that the authors know their field. Well written. But too long; approx. 750 words, including objective. Suggestions: Reduce to max 350 words.

Methodology

 Trial design: OK, but please tell us how large the main study is.

 My main concern is that I don't get what is the primary objective for this study.

1. Is it recruitment? If so, what would be regarded as a success? 1%, 2%, 5%, 10%.
2. Retention? If so, what would be regarded as a success?
3. Also you mentioned a questionnaire. What would you expect to be a success or a relevant difference?

Don't get me wrong. I do think this is important, but I think that this intervention is too weak. Or is the study explorative? I'm totally OK with that.

 In short: What would be a statistical significant and clinical relevant difference between the groups?

We confirm that we have read this submission and believe that we have an appropriate level of expertise to state that we do not consider it to be of an acceptable scientific standard, for reasons outlined above.

# Response

**Published:** 2020-03-18

**Authors:** Christopher Dwyer

**Affiliations:** NUI Galway, Ireland

Many thanks for your comments. We considered each in turn and, have amended the manuscript in light of these considerations. Other comments and questions we answer here.

**My main concern is that it is not clear what the primary outcome is? See methods.**

 All outcome measures are ‘primary’, given the exploratory nature of this non-inferiority SWAT.

**The manuscript would be much better if it could be shorter, especially the introduction.**

 The introduction has been edited to strengthen clarity and is now shorter.

**Don't use so many abbreviations.**

 This is stylistic in nature; also given that the use of acronyms enhances the flow of prose-reading and shortens the manuscripts length, we have not cut any acronyms.

**I can’t find the two participant information sheet (PIS) (am I missing something?). It would be good to find a link early in the text.**

 Please see ‘Extended data’. 

**Generalisability - can the result of this study be used in other study groups or other diagnosis?**

 Given that the intervention manipulation does not regard MS, then yes, it is somewhat generalisable. However, this reasoning is speculative and we recommend replication in other cohorts in order to observe whether there is some effect of using a MS cohort on such PIS manipulation.

**How is the cognitive status of this patients? Can this affect the result of this study or the content in the PIS?**

 Participants will have cognitive difficulties; but, consistent with the PISs (again see ‘extended data’), such difficulties would not extend to compromising ability to ‘provide informed consent’. However, given the prospective nature of this protocol, we cannot be sure that such cognitive difficulties will not affect the results of the study (let alone the protocol); thus, this comment will surely be considered in the results paper.

**Did the PPI member that were designing the PPIPS had MS so that they could relate to specific questions and feelings in the information? That part is not clear. Maybe you should describe it more thoroughly.**

 “A *PPI-designed-and-informed PIS* (PPIPIS) was developed by a PPI member of the research team - who was neither from a research or medical background, nor had experience in these fields - who would otherwise be eligible to participate in the intervention.”

**Title**

**A little wordy. Is it possible to shorten?**

**Maybe: Comparing standard information with patient-designed sheet: Protocol for a study-within-a-trial.**

 Though the title might be considered long, we believe the existing title ensures accuracy and clarity.

**Abstract**

**Background: It is a little hard to know how large the larger study is, just adding n=… would help. “The SWAT is part of a larger trial (n=…)”**

 “Methods: During the study, 120 people with multiple sclerosis…”

**Question: Are they randomly allocated 1:1?**

 Yes, amended.

**"Recruitment and retention will be analysed, as well as decision certainty, likability and understanding." Question: What is the primary outcome? How are the recruitment analysed? And I wonder, would you really expect higher recruitment and retention? I would be surprised if you get +2-3% more recruitment.**

 Recruitment is assessed dichotomously – consent or no consent. All outcome measures are ‘primary’, given the exploratory nature of this non-inferiority SWAT, which is also why it’s inappropriate to ‘expect anything’ in this context – there are no specified hypotheses.

**"Discussion: Results will provide recommendations for recruitment, consent and retention for future trials, as well as shed some light on the factors influencing the understanding and likeability of a trial’s participant information sheet." Question: This is a very strong statement. It might provide recommendations on recruitment. But also on consent, and retention?**

 The results – be they positive, negative or neutral will indeed provide information regarding manipulation of PISs and their effects on recruitment, retention, consent, etc. Of course, like all research, replication of results will be necessary; however, this SWAT will contribute to this area of study.

**Introduction**

**It is obvious that the authors know their field. Well written. But too long; approx. 750 words, including objective. Suggestions: Reduce to max 350 words.**

 The introduction has been edited to strengthen clarity and is now shorter.

**Methodology**

**Trial design: OK, but please tell us how large the main study is.**

 “Methods: During the study, 120 people with multiple sclerosis…”

**My main concern is that I don't get what is the primary objective for this study.**

 “The objective of the current research (i.e. a study-within-a-trial; SWAT) is to compare the effects of two PISs designed to facilitate informed consent of potential participants – a (patient) PPI-designed-and-informed PIS in comparison and a standard, researcher-designed PIS on: recruitment, decision certainty, retention; understanding, readability, accessibility, likeability and decision to consent.”

**Is it recruitment? If so, what would be regarded as a success? 1%, 2%, 5%, 10%.**

 With respect to the primary outcomes, again all outcome measures are ‘primary’, given the exploratory nature of this non-inferiority SWAT. We are not concerned with feasibility standards in this SWAT, such as overall recruitment success. The SWAT is a comparison study.

**Retention? If so, what would be regarded as a success?**

 See response directly above.

**Also you mentioned a questionnaire. What would you expect to be a success or a relevant difference?**

**Don't get me wrong. I do think this is important, but I think that this intervention is too weak. Or is the study explorative? I'm totally OK with that.**

 Again, the study is exploratory. A ‘relevant difference’ will be determined at the .05 level of statistical significance. This has been added for clarity.

**In short: What would be a statistical significant and clinical relevant difference between the groups?**

 See above.

 With respect to the reports regarding the rationale, objectives, design, methods and datasets, we hope our responses have provided clarity and answer the questions arising from your review.

# Peer review: Reviewer response for version 1

**DOI:** 10.21956/hrbopenres.14067.r27170

**Published:** 2020-03-04

**Authors:** Patricia M. Kearney, Emmy Racine

**Affiliations:** 1 School of Public Health, University College Cork, Cork, Ireland

**Overall comments**

 Thank you for the opportunity to review this protocol. I have conducted this review under the supervision of Professor Patricia Kearney, School of Public Health, University College Cork. The protocol describes the study rationale and design of a Study Within A Trial (SWAT). This SWAT will be conducted within a larger trial that aims to evaluate the feasibility and preliminary efficacy of a cognitive occupation-based programme for people with MS (COB-MS). The purpose of the SWAT is to compare a PPI designed Patient Information Sheet (PPIPIS) to a standard, researcher-designed Patient Information Sheet (SRPIS). The outcome measures include trial recruitment and retention along with decision certainty, understanding, readability, accessibility, likeability and decision to consent.

 The study addresses a relevant and important research question. Although, PPI is increasingly emphasized as an essential component of health research, evidence on the impact of PPI is piecemeal and inconclusive.

 Comments by section:

**Abstract**

 Background: This paragraph provides the reader with a summary of the SWAT instead of providing background on the study context. I suggest rephrasing this paragraph to include the key points from each of your paragraphs in the introduction section of your paper. Then state the study objective. This will clarify what the knowledge gap is and how you aim to address it.

 Methods: How will decision certainty, likeability and understanding be analyzed? What are the data collection and analysis procedures?

 Discussion: As is, this paragraph does not reflect the same points that you make in the discussion section of the paper. I suggest rephrasing this paragraph to reflect the key points that you put forward in the discussion.

**Introduction**

 In the introduction, the authors draw on a range of literature and the study objective is clear. However, the introduction is quite long. You have included the key information that the reader needs to know but there is some overlap between paragraphs and the knowledge gap you are trying to address could be made more explicit. I suggest you re-phrase the Introduction so that each paragraph outlines the key points that the reader needs to know (e.g. PIS, Why PIS are important, PPI, What we know about PPI (e.g. Cockayne *et al*. ^1^ , Crocker *et al*. ^2^), what we don’t know about PPI- knowledge gap, study objective). Some of the references to existing literature could be moved to the discussion.

 Other suggestions for the Introduction:

- Paragraph 1: ‘primary source of written information’- I suggest inserting the word ‘written’ here as potential participants often receive verbal information from the study team before they consent to participate.
- Paragraph 1: What do you mean by ‘quality’ here? Do you mean easy to understand?
- Provide SWAT definition/explanation and explain to the reader why SWATs are important.

**Methods**

 Ethical statement: Word missing from first sentence, suggest adding- ‘ the study will be conducted at the….’

 Trial design: decision to consent is mentioned twice in the first sentence.

 Intervention: As it is currently written, you explain the SRPIS first and then describe the PPIPIS. I think this order makes sense but in the rest of the paper (including study objective) you refer to the PPIPIS first and SRPIS second. Please ensure that the order is consistent throughout the paper.

 Outcome measures: The six-item questionnaire is not an objective measure of readability, understanding, likeability etc. I understand that this questionnaire cannot be changed as study recruitment has already started but I think you need to justify why you did not choose an objective measure and highlight in your discussion that this is a limitation of the study.

 Procedure: Rephrase 5 ^th^ sentence, it is currently too long and difficult to understand. Also, will the same researcher phone all potential participants? This is important as previous studies have shown that the researchers’ manner/approach can influence the persons decision to participate.

 PPI: Suggest moving the definition of PPI to the Introduction and keeping this paragraph as an explanation of the PPI activities within this particular study.

**Discussion**

 The discussion should include:

- The strengths and limitations of the study. You don’t need to go into too much detail but show that you are aware (i.e. strength-important research question that adds to the evidence base for PPI, limitation- six-item questionnaire is not an objective measure).
- Add some more information on the transferability of findings. You mention that the current SWAT protocol could provide a template for future clinical research in MS. Is the PPI-PIS designed specifically for use with PwMS or is there potential for it to be adapted in studies with other populations?

**Dissemination:**

 The knowledge exchange plan is well thought out. The results of this SWAT will be of interest to members of the research community (especially those interested in PPI), PPI contributors and funding bodies. Well done!

We confirm that we have read this submission and believe that we have an appropriate level of expertise to confirm that it is of an acceptable scientific standard.

## References

[1] : Guidelines for reporting embedded recruitment trials. *Trials*.2016;17: 10.1186/s13063-015-1126-y 27 10.1186/s13063-015-1126-y 26767365PMC4714476

[2] : Impact of patient and public involvement on enrolment and retention in clinical trials: systematic review and meta-analysis. *BMJ*.2018;363: 10.1136/bmj.k4738 k4738 10.1136/bmj.k4738 30487232PMC6259046

# Response

**Published:** 2020-03-18

**Authors:** Christopher Dwyer

**Affiliations:** NUI Galway, Ireland

Many thanks for your comments. We considered each in turn and, have amended the manuscript in light of these considerations. Other comments and questions we answer here.

**Abstract**

**Background: This paragraph provides the reader with a summary of the SWAT instead of providing background on the study context. I suggest rephrasing this paragraph to include the key points from each of your paragraphs in the introduction section of your paper. Then state the study objective. This will clarify what the knowledge gap is and how you aim to address it. Methods: How will decision certainty, likeability and understanding be analyzed? What are the data collection and analysis procedures? Discussion: As is, this paragraph does not reflect the same points that you make in the discussion section of the paper. I suggest rephrasing this paragraph to reflect the key points that you put forward in the discussion.**

The Abstract has been amended to more accurately reflect the Background section. Given the length of the response regarding methods in the abstract, we have chosen to leave such discussion until the outcome measures and analysis sections. Finally, the discussion section of the Abstract has been amended to include further implications of the SWAT; and as a result, the Abstract and Discussion are now consistent.

**Introduction**

**In the introduction, the authors draw on a range of literature and the study objective is clear. However, the introduction is quite long. You have included the key information that the reader needs to know but there is some overlap between paragraphs and the knowledge gap you are trying to address could be made more explicit. I suggest you re-phrase the Introduction so that each paragraph outlines the key points that the reader needs to know (e.g. PIS, Why PIS are important, PPI, What we know about PPI (e.g. Cockayne *et al*.** **^1^** **, Crocker *et al*.** **^2^), what we don’t know about PPI- knowledge gap, study objective). Some of the references to existing literature could be moved to the discussion.**

The introduction has been edited to strengthen clarity and is now shorter.

**Other suggestions for the Introduction:**

****Paragraph 1: ‘primary source of written information’- I suggest inserting the word ‘written’ here as potential participants often receive verbal information from the study team before they consent to participate.**** 

‘Written’ has been added to ‘primary source of information’ to enhance clarity. 

**Paragraph 1: What do you mean by ‘quality’ here? Do you mean easy to understand?** 

Yes, by quality, we mean easy to understand; but, we also mean readability and likeability – other factors considered throughout the protocol. However, it would not be appropriate to introduce these concepts yet at this point, from the standpoint of ensuring logical ‘flow’.

**Provide SWAT definition/explanation and explain to the reader why SWATs are important.** 

In an effort to keep the introduction as succinct and concise as possible, we chose not to define or elaborate on SWAT – we deemed explanation of the acronym sufficient for the protocol.

**Methods**

**Ethical statement: Word missing from first sentence, suggest adding- ‘ the study will be conducted at the….’**

Amended.

**Trial design: decision to consent is mentioned twice in the first sentence.**

Amended.

**Intervention: As it is currently written, you explain the SRPIS first and then describe the PPIPIS. I think this order makes sense but in the rest of the paper (including study objective) you refer to the PPIPIS first and SRPIS second. Please ensure that the order is consistent throughout the paper.**

Amended.

**Outcome measures: The six-item questionnaire is not an objective measure of readability, understanding, likeability etc. I understand that this questionnaire cannot be changed as study recruitment has already started but I think you need to justify why you did not choose an objective measure and highlight in your discussion that this is a limitation of the study.**

Given that readability, understanding and likeability are different from person to person, self-report was deemed the most appropriate method of assessing these. Though these are subjective from the participants’ perspectives (as are all self-report measures) the measure is objective from a data analysis perspective (i.e. through examination of a quantitative measure). With respect to using an *established* measure, the DCS was the only measure known to our research group that addressed our targeted outcomes. The questionnaire was purpose-built to address other outcomes of interest.

**Procedure: Rephrase 5 ^th^ sentence, it is currently too long and difficult to understand. Also, will the same researcher phone all potential participants? This is important as previous studies have shown that the researchers’ manner/approach can influence the persons decision to participate.**

Amended.

**PPI: Suggest moving the definition of PPI to the Introduction and keeping this paragraph as an explanation of the PPI activities within this particular study.**

Amended.

**Discussion**

**The discussion should include:**

**The strengths and limitations of the study. You don’t need to go into too much detail but show that you are aware (i.e. strength-important research question that adds to the evidence base for PPI, limitation- six-item questionnaire is not an objective measure).**

As this is a protocol, we have deemed this inappropriate for this manuscript - given the prospective, exploratory nature of this non-inferiority SWAT, we are as of yet unaware of its strengths and weaknesses. Upon conducting the study and applying the protocol, strengths and weaknesses will be discussed, alongside study findings.

**Add some more information on the transferability of findings. You mention that the current SWAT protocol could provide a template for future clinical research in MS. Is the PPI-PIS designed specifically for use with PwMS or is there potential for it to be adapted in studies with other populations?** 

Amended – implications of MS-specific PIS manipulations are now addressed (but not thoroughly elaborated, given the prospective nature of the protocol).

**Dissemination:**

**The knowledge exchange plan is well thought out. The results of this SWAT will be of interest to members of the research community (especially those interested in PPI), PPI contributors and funding bodies. Well done!**

Many thanks for your comments!

# Peer review: Reviewer response for version 1

**DOI:** 10.21956/hrbopenres.14067.r27122

**Published:** 2020-02-24

**Authors:** Vivian Welch

**Affiliations:** 1 School of Epidemiology and Public Health, Faculty of Medicine, University of Ottawa, Ottawa, Canada

This is a well-written, well-reasoned and clear protocol for a study within a trial to assess whether a patient/public designed participant information sheets is preferred by participants and/or leads to better retention for a clinical trial.

 The methods are well-described in sufficient detail for replication. The protocol is available on the open science framework already and anonymized data will be made available according to FAIR principles.

 My only comment in writing up the trial is that the rationale for the decisional conflict scale to focus on the SWAT rather than the main trial was not clear until later in the protocol, where the authors state that since eligibility is assessed after the SWAT.

I confirm that I have read this submission and believe that I have an appropriate level of expertise to confirm that it is of an acceptable scientific standard.

# Response

**Published:** 2020-03-18

**Authors:** Christopher Dwyer

**Affiliations:** NUI Galway, Ireland

Many thanks for your comments.
